# Typhoid Fever at Lincoln

**Published:** 1905-03-04

**Authors:** 


### The Hospital.

A Journal of

The Medical Sciences and Hospital Administration.

Vol. XXXVII.—No. 962. MARCH 4, 1905.

### Typhoid Fever at Lincoln.

It seems not wholly unreasonable to hope that the epidemic of typhoid fever still running its course at Lincoln may ultimately be contributable to the public safety. Similar occurrences, although happily not upon so large a scale, are by no means infrequent; but the lessons which they are calculated to convey are too often lost by reason of the time which is suffered to elapse before they are impressed upon the minds of those by whom they are chiefly needed. An outbreak occurs in some country town or in some rural district; and the facts relating to it, the conditions which permitted its commencement and its diffusion, are for the first time given to the public some eighteen months after the event, in the next annual report of the medical officer of the Local Government Board, or, in other words, in a publication possessing little attractiveness of form, and reaching only a comparatively small circle of readers. The events related are already ancient history, and they scarcely receive notice. The victims of the epidemic are dead and buried, the survivors have either returned to their places in the ranks of life, or, broken down by complications or sequelæ, have dropped out of them and are forgotten, and the "local authorities" concerned are careful to avoid the initiation of reforms which would only serve, in their opinion, to recall attention to their former neglects and omissions. In the case of Lincoln the iron has been struck while it was hot. A "special correspondent" of the *Times* has investigated the facts as they have occurred, and has given the results of his investigations to the world. From his narrative, which appeared in the issues of that paper for February 23rd and 25th, it seems that the epidemic commenced on December 2nd, and that down to February 18th 680 cases had been notified, with, still up to that time only, 55 deaths. It is further reported that the type of fever is "peculiar," and that it is marked by a persistent and obstinate high temperature, which continues for as long as 30 or 35 days, and which certainly points to a probability of dangerous sequelæ. As far as can be ascertained, the epidemic is already on the decline, only 106 cases having been notified during the week ending February 18th, against 154 in the week ending on the 11th, and 268 during the week ending on the 4th.

The cause of the outbreak is fortunately absolutely clear, and seems to admit of no discussion or dispute. The water supply of the city is derived from gathering grounds which are exposed to every kind of excremental pollution, and to the character and dangers of which attention was called 20 years ago. In a report of that date, Dr. Harrison, the medical officer of the city, showed that the water, after filtration, contained an excess of organic matter, that its sources were polluted by drainage from land highly cultivated and occasionally manured with night-soil, and that the quantity available was not sufficient for the wants of the inhabitants. The whole question then entered upon the stage of "discussion," a process to which the Local Government Board contributed more than one condemnation of the supply, while a newly established sewage farm, connected with the Bracebridge Asylum, "more embroiled the fray" by contributing its effluent to the river Witham above the intake. Negotiations for excluding this last contribution were set on foot, but were abandoned when it was discovered that they could not be rendered fruitful at a smaller cost than £15. In 1898 a deep boring in the sandstone was determined upon, and was actually commenced in 1891, but in two years, when it had been carried to a depth of 880 feet, it terminated in a breakdown. Something gave way, the boring tool was lost, and the rock caved in above it. A fresh stage of "discussion" was then entered upon, and so the years rolled by. Of the immediate cause of the actual catastrophe nothing has so far been made public, but, if the facts were known, it would probably be traceable to some single case of typhoid, perhaps in the person of some passing vagrant, whose excreta were contributed to the general mass of the ordinary daily pollution. The case is supposed to have been aggravated by the practice, which seems to have been general in the city, of allowing the taps to run during the continuance of frost. By reason of this practice, the consumption of water rose in November by 50 per cent., and the rate of filtration was increased to meet the demand. The accepted rate of filtration is 450 gallons to the square yard of filtering area, and at Lincoln, from November 29th to January 14th, it was going on at the rate of 750 gallons to the square yard, and so might easily fail to arrest matters which would have been arrested at the ordinary rate. This, however, is hardly enough to explain the epidemic, and it is necessary to remember, in assigning any blame for the event, that the typhoid bacillus ought not to have been there for the filter beds to arrest. Still, with such arrangements as those of Lincoln, a serious outbreak was manifestly certain to occur sooner or later, and the actual cause of it was neither more nor less than the absolute unfitness of the members of the corporation, or whatever the local authority may be called, to deal effectively with the responsibilities which their position entailed upon them. It is the curse of England that, because a man has shown decent character and fair capacity in one direction, we immediately seize upon him to do important work in another. The members of the Lincoln corporation have, no doubt, gained the esteem of their fellow citizens as honourable and successful men of business; and, on no better ground than this, and with no other qualification than pure ignorance, they are permitted to be responsible for the public health of their locality. Nero fiddled while Rome was burning, and the Lincoln corporation have "discussed" for twenty years with typhoid fever at their doors. At last it has come, and the only hope permitted by the circumstances is that the lessons which it is calculated to teach may not be wholly thrown away upon those whom they most concern.

